# Supplementary material for: Polymorphism of the Myostatin (MSTN) Gene in Landes and Kielecka Geese Breeds
Source: Animals (Basel). 2019 Dec 19;10(1):10. doi: 10.3390/ani10010010 (PMC7022763; doi:10.3390/ani10010010)
Supplement: Supplementary file 1 [file animals-10-00010-s001.pdf]

Table S1: Nutrient contents of feed mixtures

Item (% kg of all-mash)	Age of geese in weeks		
	1-10	11-12	13-95
Crude protein	18.5	14.5	14.75
Crude fat and oils*	2.3	2.3	2.34
Crude fibre	4.5	4.5	4.8
Crude ash	4.9	4.9	9.62
Methionine	0.41	0.32	0.25
Lysine	0.8	0.63	0.6
Calcium	1.5	0.64	2.49
Total Phosphorus	1	0.32	0.33
Natrium	0.16	0.15	0.16
Vit. A (IU/kg)	10 000	10 000	10 000
Vit. D3 (IU/kg)	3 000	2 500	3 000
Vit. E (mg/kg)	35.2	21	50

* only in group in age of 13-95 week of life
